# Sterbeorte von COVID-19-Patienten: eine Observationsstudie auf Grundlage ausgewerteter Todesbescheinigungen der Stadt Münster (2021)

**DOI:** 10.1007/s00103-023-03702-7

**Published:** 2023-05-26

**Authors:** Lukas Manglus, Philipp Lenz, Burkhard Dasch

**Affiliations:** Zentrale Einrichtung Palliativmedizin, Universitätsklinikum Münster, Albert-Schweitzer-Campus 1, Gebäude W30, 48149 Münster, Deutschland

**Keywords:** COVID-19-Sterbefälle, SARS-CoV‑2, Sterbeorte, Todesbescheinigungen, Observationsstudie, COVID-19 deaths, SARS-CoV‑2, Places of death, Death certificates, Observational study

## Abstract

**Hintergrund:**

Die Sterbeorte von COVID-19-Patienten wurden in Deutschland bislang nur wenig untersucht.

**Methoden:**

In einer westfälischen Sterbeortstudie wurden in der Stadt Münster anhand sämtlicher Todesbescheinigungen von 2021 statistische Auswertungen durchgeführt. Personen, die mit oder an einer COVID-19-Erkrankung verstorben waren, wurden anhand der ärztlichen Angaben zur Todesursache identifiziert und mit deskriptiv statistischen Verfahren unter Anwendung von SPSS analysiert.

**Ergebnisse:**

Es wurden insgesamt 4044 Todesbescheinigungen ausgewertet. Hierbei wurden 182 verstorbene COVID-19-Patienten identifiziert (4,5 %). Bei 159 Personen (3,9 %) war die COVID-19-Erkrankung todesursächlich, wobei deren Sterbeorte sich wie folgt verteilten: Krankenhaus insgesamt 88,1 % (Intensivstation 57,2 %, Palliativstation 0,0 %), Hospiz 0,0 %, Pflegeheim 10,7 %, zu Hause 1,3 %, sonstiger Ort 0,0 %. Erkrankte < 60 Jahre verstarben zu 100 % im Krankenhaus, über 80-Jährige in 75,4 % der Fälle. Nur 2 COVID-19-Patienten, die jeweils über 80 Jahre alt waren, verstarben zu Hause. Sterbefälle im Pflegeheim (17) betrafen mehrheitlich ältere Frauen. 10 dieser Heimbewohner waren von einem spezialisierten ambulanten Palliativteam am Lebensende betreut worden.

**Diskussion:**

COVID-19-Erkrankte verstarben überwiegend im Krankenhaus. Fulminante Krankheitsverläufe mit hoher Symptomlast und das nicht selten junge Alter der Erkrankten sind hierfür potenzielle Erklärungsansätze. Stationäre Pflegeeinrichtungen spielten als Sterbeort bei lokalen Infektionsausbrüchen eine gewisse Rolle. Erkrankte Personen verstarben nur selten zu Hause. Infektionsschutzmaßnahmen können ein Grund dafür sein, dass keine Patienten in Hospizen oder auf Palliativstationen verstarben.

**Zusatzmaterial online:**

Zusätzliche Informationen sind in der Online-Version dieses Artikels (10.1007/s00103-023-03702-7) enthalten.

## Abstract (en)

**Introduction:**

The places of death of COVID-19 patients have so far hardly been investigated in Germany.

**Methods:**

In a places of death study in Westphalia (Germany), statistical evaluations were carried out in the city of Muenster on the basis of all death certificates from 2021. Persons who had died with or from a COVID-19 infection were identified by medical information on cause of death and analyzed with descriptive statistical methods using SPSS.

**Results:**

A total of 4044 death certificates were evaluated, and 182 deceased COVID-19 patients were identified (4.5%). In 159 infected patients (3.9%), the viral infection was fatal, whereby the distribution of places of death was as follows: 88.1% in hospital (57.2% in the intensive care unit; 0.0% in the palliative care unit), 0.0% in hospice, 10.7% in nursing homes, 1.3% at home, and 0.0% in other places. All infected patients < 60 years and 75.4% of elderly patients ≥ 80 years died in hospital. Only two COVID-19 patients, both over 80 years old, died at home. COVID-19 deaths in nursing homes (17) affected mostly elderly female residents. Ten of these residents had received end-of-life care from a specialized outpatient palliative care team.

**Discussion:**

The majority of COVID-19 patients died in hospital. This can be explained by the rapid course of the disease with a high symptom burden and the frequent young age of the patients. Inpatient nursing facilities played a certain role as a place of death in local outbreaks. COVID-19 patients rarely died at home. Infection control measures may be one reason why no patients died in hospices or palliative care units.

## Hintergrund

Nach der weltweiten Ausbreitung des Coronavirus SARS-CoV‑2 im Frühjahr 2020 folgten tiefgreifende Veränderungen des gesellschaftlichen Lebens [1]. Allein bis zum Jahr 2022 infizierten sich weltweit über 280 Mio. Menschen. Mehr als 5,4 Mio. an COVID-19 erkrankte Personen verstarben. In Deutschland wurden 2020–2021 über 7,16 Mio. Infizierte und 114.521 Todesfälle (2020: 41.647, 2021: 72.874) gemeldet. Zumindest zeitweise wurde eine Übersterblichkeit festgestellt [2–4].

Bestimmte Einflussfaktoren (hohes Alter, Adipositas, chronische Lungenerkrankung, Multimorbidität) sind mit einer erhöhten Wahrscheinlichkeit für eine Krankenhausaufnahme und für ein Versterben von COVID-19-erkrankten Personen assoziiert [5]. Dennoch sind auch jüngere Menschen ohne Vorerkrankungen von einem schweren Krankheitsverlauf und Versterben betroffen [6]. Zudem sind tödliche Verläufe bei infizierten Schwangeren bekannt [7].

Bis zur Zulassung der ersten Impfstoffe im Dezember 2020 stellten nichtpharmazeutische Interventionen wie Social Distancing, Lockdowns und Quarantäne- bzw. Isolationsmaßnahmen die einzigen effektiven Schutzmaßnahmen dar [8–10]. Dies stellte behandelnde Ärzte vor ungekannte Herausforderungen. So musste bei hoch vulnerablen Patientengruppen sogar teilweise abgewogen werden, ob Sterbebegleitung aufgrund restriktiver Infektionsschutzmaßnahmen überhaupt stattfinden kann [11, 12]. Für COVID-19-Patienten hatte dies insbesondere zu Beginn der Pandemie schwere Folgen. Aufgrund von strengen Isolationsmaßnahmen und zu wenig vorhandener Schutzausrüstung wurde beobachtet, dass auch in der letzten Lebensphase Besuche von Angehörigen, Freunden und in der Palliativversorgung Mitarbeitenden (Palliativdienste, Therapeuten, Seelsorger, Ehrenamtspersonen) nur sehr eingeschränkt stattfanden. Der Prozess des Abschiednehmens war dadurch für Sterbende und Hinterbliebene erschwert bis teilweise unmöglich. Ein vermehrter Einsatz digitaler Medien zur Videotelefonie und Telemedizin konnten dies nur beschränkt mildern. Das im Rahmen des Netzwerks Universitätsmedizin im August 2020 ins Leben gerufene Projekt „Palliativversorgung in Pandemiezeiten“ (PallPan) beschäftigte sich wissenschaftlich mit den Herausforderungen und gab Handlungsempfehlungen für verschiedene Beteiligte (Patienten, Angehörige, medizinisches Personal) heraus [13]. In den späteren Pandemiephasen wurden bisherige restriktive Besuchsregelungen bei infizierten Sterbenden durch den Nachweis eines negativen SARS-CoV-2-Nasen-Rachen-Abstriches für Angehörige gelockert [14].

In Deutschland erfolgte eine zeitlich wellenförmige Ausbreitung des Virus [15]. Große Kliniken mit entsprechender technischer Ausstattung und Intensivkapazitäten nahmen in den Hochphasen der Pandemie eine Sonderrolle ein. So wurden Patienten aus anderen Regionen, teilweise länderübergreifend dorthin verlegt. In Zusammenarbeit von Robert Koch-Institut (RKI) und Deutscher Interdisziplinärer Vereinigung für Intensiv- und Notfallmedizin (DIVI) wurde ein Intensivregister zur Echtzeitüberwachung der bundesweiten Kapazitäten angelegt [16]. In speziell eingerichteten Krankenhäusern der Schwerpunkt- und Maximalversorgung war es möglich, die extrakorporale Membranoxygenierung (ECMO) als therapeutische Ultima Ratio bei COVID-19-Patienten mit schwerstem respiratorischen Krankheitsverlauf anzuwenden. Die Krankenhaussterblichkeit unter dieser Therapie betrug 37,1 % (95 %-Konfidenzintervall (KI): 32,3–42,0 %; [17]). In der Studienregion der Stadt Münster existierten 3 Krankenhäuser mit ECMO-Therapieplätzen, sodass an diesen medizinischen Zentren schwerstkranke COVID-19-Patienten – zum Teil auch aus anderen Bundesländern und Nachbarländern (Niederlande, Belgien) – zugewiesen wurden [18].

Der Sterbeort ist in der Palliativmedizin ein wichtiger Indikator für die Versorgungsqualität am Lebensende. Repräsentativen Untersuchungen zufolge wünschen sich die meisten Menschen in Deutschland zu Hause zu versterben [19]. Andererseits tendieren ältere und morbide Menschen häufiger als junge, gesunde Personen dazu, in einer Institution wie dem Krankenhaus, dem Pflegeheim oder dem Hospiz versterben zu wollen.

Die tendenzielle Entwicklung der Sterbeorte in Deutschland im Verlauf der letzten Jahrzehnte wurde auf Grundlage ausgewerteter Todesbescheinigungen u. a. in 4 Regionen Westfalens eingehend erforscht (Städte Bochum und Münster, Landkreise Borken und Coesfeld; Nordrhein-Westfalen (NRW); [20, 21]). Mit über 40.000 ausgewerteten Todesbescheinigungen stellt die westfälische Sterbeortstudie eine der umfangreichsten Untersuchungen zu diesem Thema in Deutschland dar. Dabei erstreckten sich die Analysen über einen 20-jährigen Beobachtungszeitraum (2001 bis 2021). Es zeigt sich, dass die meisten Menschen im Krankenhaus versterben. Pflegeheime und für die Sterbebegleitung spezialisierte Einrichtungen (Hospize, Palliativstationen) nehmen als Sterbeorte kontinuierlich an Bedeutung zu. Andererseits ereignen sich immer weniger Sterbefälle zu Hause.

Bislang ist der Sterbeort von verstorbenen COVID-19-Patienten in Deutschland nur wenig untersucht. Es existieren Studienergebnisse von Gleich et al., einer Forschungsgruppe des Gesundheitsreferates München, die im Stadtgebiet der bayerischen Landeshauptstadt eine Untersuchung auf Basis analysierter Todesbescheinigungen durchführten. Im Zeitraum zwischen März und Dezember 2020 wurden insgesamt 1029 gesicherte COVID-19-Sterbefälle identifiziert. Die Erkrankten verstarben mit 71,8 % in der überwiegenden Mehrzahl im Krankenhaus, gefolgt vom Pflegeheim (24,4 %) und zu Hause (3,8 %; [22]). Eine weitere Sterbeortuntersuchung wurde im Landkreis Gießen im Zeitraum 11/2020 bis 04/2021 durchgeführt [23]. Todesbescheinigungen von 471 COVID-19-Sterbefällen wurden retrospektiv ausgewertet. Hierbei wurde folgende Sterbeortverteilung ermittelt: 63,5 % Krankenhaus, 34,0 % Pflegeheim, 2,1 % zu Hause, 0,4 % sonstiger Ort.

Daten aus dem Vereinigten Königreich zeigen interessanterweise, dass in den ersten Pandemiemonaten mehr Menschen mit schweren Erkrankungen wie bösartigem Tumorleiden oder Demenz zu Hause verstarben als zuvor. Die Autoren der Registerstudie führen dies darauf zurück, dass viele Patienten die Krankenhäuser während der Zeit des Lockdowns mieden [24]. Auch in Deutschland verzeichnete man beispielsweise bei kardiologischen Notfällen einen Rückgang der Krankenhausaufnahmen [25, 26].

Im Jahr 2021 wurde in der Stadt Münster eine Follow-up-Erhebung der westfälischen Sterbeortstudie durchgeführt. Vor dem Hintergrund der anhaltenden SARS-CoV-2-Pandemie stellte sich die Frage, wie der Sterbeort von COVID-19-Patienten verteilt war. Die Ergebnisse der Studie werden nachfolgend im Detail aufgeführt.

## Methoden

### Studiendesign, Datengrundlage.

Bei der folgenden Arbeit handelt es sich um eine epidemiologische Querschnittsstudie. Grundlage der Studie bildeten sämtliche ärztliche Todesbescheinigungen der Stadt Münster des Jahres 2021. Der Beobachtungszeitraum erstreckte sich vom 01.01.2021 bis 31.12.2021.

Da den Gesundheitsämtern die Aufbewahrungspflicht des nichtvertraulichen und vertraulichen Teils der Todesbescheinigung obliegt [27], wurden für die vorliegende Untersuchung im Gesundheitsamt Münster archivierte Todesbescheinigungen in einer Datenbank (Excel-Liste) erfasst, in ein spezielles Statistikprogramm übertragen und anschließend deskriptiv ausgewertet.

### Feststellung des Sterbeortes.

Anhand des Textfeldes „Sterbeort“ und der angegebenen Adresse in der Todesbescheinigung wurden die Daten den Kategorien zu Hause, Krankenhaus (hier weiter unterschieden nach Intensivstation und Palliativstation), Pflegeheim, Hospiz und sonstiger Ort zugeordnet. Die Feststellung des Sterbeortes erfolgte anhand der niedergeschriebenen Sterbeortanschrift bzw. anhand des Institutsstempels.

### Todesursache

Die Todesursache wird in der Todesbescheinigung des Landes NRW in Freitextfeldern anhand der Kausalkette vom ursächlichen Grundleiden bis zur unmittelbaren Todesursache dokumentiert. Hinzu kommen die zusätzlichen „mit zum Tode führenden Erkrankungen“ sowie ein Textfeld für die Epikrise [27]. In der Studie wurde jegliche ärztliche Information zur Todesursache erfasst, wobei ein besonderes Augenmerk auf bösartige Tumorerkrankungen (Internationale statistische Klassifikation der Krankheiten, ICD-10: C00–96) und auf Demenzerkrankungen (ICD-10: F01, F02, F03, G30) gelegt wurde.

#### Identifizierung COVID-19-Verstorbener.

Die ärztlichen Angaben zur Todesursache wurden nach bestimmten Schlüsselwörtern abgesucht: COVID, COVID-19, SARS-CoV‑2, Corona. Auch wurde auf die Dokumentation einer Pneumonie, eines akuten Atemnotsyndroms (ARDS), einer erfolgten nichtinvasiven oder invasiven Beatmung sowie einer ECMO-Anwendung geachtet. Anhand dieser Informationen wurden COVID-19-Verstorbene identifiziert und in 2 Gruppen eingeteilt: a) Personen mit todesursächlicher COVID-19-Erkrankung; b) Personen mit COVID-19 als Begleiterkrankung.

#### Auswertungsmethoden.

Die Daten wurden statistisch deskriptiv analysiert. Nominale Variablen wurden in absoluten und relativen Anteilen dargestellt, stetige Variablen durch die Maßzahlen Mittelwert, Median, Minimum und Maximum.

Beim Sterbeort Krankenhaus war anhand des Institutsstempels nicht immer in die Sterbeorte Intensiv- und Palliativstation differenzierbar. Aus diesem Grund wurde eine pseudonymisierte Recherche mit Hilfe des Medizincontrollings aller Münsteraner Kliniken durchgeführt.

Die Differenzierung in „mit“ oder „an“ COVID-19 verstorbenen Personen ließ sich mehrheitlich gut durchführen. Lag in Einzelfällen eine strittige Beurteilung vor, wurde ein verblindeter ärztlicher Expertenabgleich von den Autoren (BD, LM) durchgeführt und nach ausführlicher Diskussion eine gemeinsame Entscheidung getroffen, um möglichst höchste Plausibilität der Daten zu erlangen. Zudem wurde ein pseudonymisierter Abgleich zwischen im häuslichen Umfeld oder im Pflegeheim verstorbenen COVID-19-Patienten mit Daten des lokalen ambulanten palliativmedizinischen Konsiliardienstes Münster (PKD Münster 2 GbR) durchgeführt. So ließ sich ermitteln, ob eine spezialisierte ambulante Palliativversorgung (SAPV) bei diesen Verstorbenen am Lebensende erfolgt war.

Statistisch getestet wurde, ob sich mit und an COVID-19 verstorbene Patienten sowie COVID-19-Verstorbene und Verstorbene ohne Hinweis auf Vorliegen dieser Infektionserkrankung in Bezug auf Variablen, wie u. a. das Alter, das Geschlecht und Begleiterkrankungen, voneinander unterschieden. Ebenso wurde bei Tumorerkrankten und Personen mit Demenz getestet, ob sich die Sterbeorte mit ihren prozentualen Anteilen zwischen 2017 und 2021 statistisch unterschieden. Hierzu wurden folgende Testverfahren herangezogen: Chi-Quadrat-Test (kategorielle Variablen), unverbundener t‑Test (stetige, normalverteilte Daten), Mann-Whitney-U-Test (stetige, nicht normalverteilte Daten). Das Signifikanzniveau wurde bei *p* < 0,05 festgelegt. Die statistische Analyse erfolgte mittels der Statistiksoftware IBM SPSS Version 28.

## Ergebnisse

### COVID-19-Fälle der Stadt Münster und die Charakteristika der verstorbenen Patienten

In der Stadt Münster lebten zum Stichtag 31.12.2021 insgesamt 317.713 Menschen (Frauen: 165.198, Männer: 152.515). Das Alter der Einwohner verteilte sich hierbei wie folgt: < 18 Jahre: 14,9 %; 18–64 Jahre: 67,4 %; ≥ 65 Jahre: 17,7 % [28].

Im Beobachtungsjahr 2021 wurden 10.858 SARS-CoV-2-Neuinfektionen labortechnisch nachgewiesen (Jan: 788; Feb: 329; Mär: 900; Apr: 1326; Mai: 408; Jun: 60; Jul: 209; Aug: 727; Sep: 736; Okt: 717; Nov: 1942; Dez: 2716; Abb. 1; [29]). Schwerpunktmäßig waren Menschen jüngeren Alters betroffen, mit einem Peak bei den 20- bis 29-Jährigen. In 2,1 % der Fälle waren ältere Menschen ≥ 80 Jahre infiziert. Die meisten COVID-19-Todesfälle ereigneten sich im Januar 2021 (Abb. 2). In diesem Monat wurden allein 58 Sterbefälle registriert.

**Figure 1:**
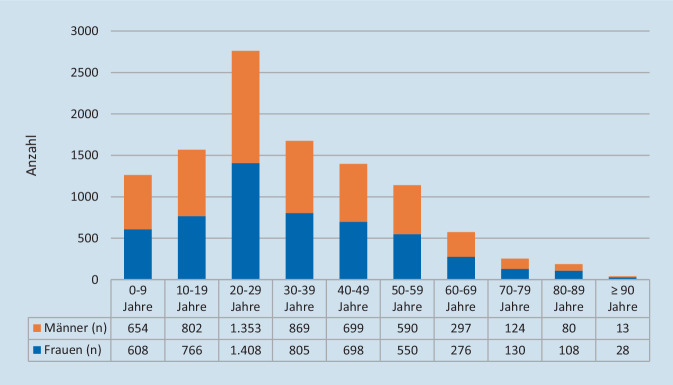

**Figure 2:**
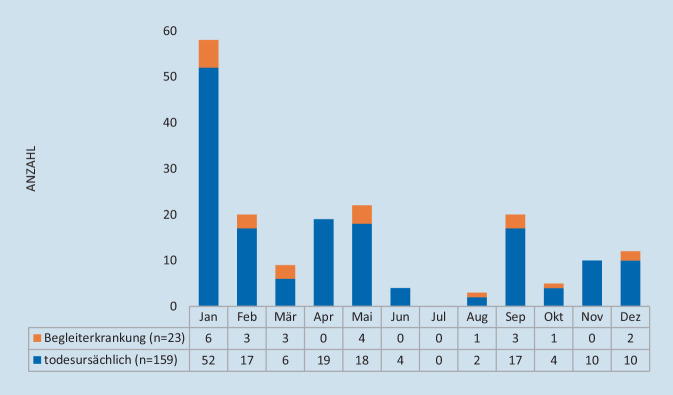

Insgesamt verstarben 4044 Menschen in der Stadt Münster, davon 182 Personen im Zusammenhang mit einer COVID-19 Erkrankung entsprechend einem Anteil von 4,5 % (Tab. 1). Bei 159 COVID-19-Patienten war die Viruserkrankung todesursächlich. Hieraus ergab sich eine Prävalenz von 3,9 % (95 %-KI: 3,3–4,5 %) bezogen auf alle Sterbefälle der Stadt. Bei 23 Personen war die nachgewiesene Viruserkrankung als Begleiterkrankung einzustufen.

**Table 1:** 

Parameter	COVID-19 todesursächlich	COVID-19 Begleiterkrankung	*p*	Sterbefälle mit COVID-19	Sterbefälle ohne COVID-19	*p*
	*n* = 159 (100 %)	*n* = 23 (100 %)		*n* = 182 (100 %)	*n* = 3862 (100 %)	
*Geschlecht*						
Männlich	94 (59,1 %)	12 (52,2 %)	0,652	106 (58,2 %)	1961 (50,8 %)	0,050^*^
Weiblich	65 (40,9 %)	11 (47,8 %)	0,652	76 (41,8 %)	1899 (49,2 %)	0,050^*^
*Sterbealter, Jahre*						
Mittelwert	71,9	73,5	0,676	72,1	76,6	0,001^*^
Median	75,7	78,3	–	75,7	80,9	–
Min./Max.	28,6/101,3	4,6/99,8	–	4,6/101,3	0,0/104,9	–
*Altersgruppen*						
0–19 Jahre	0 (0,0 %)	1 (4,3 %)	0,008^*^	1 (0,5 %)	66 (1,7 %)	0,095
20–39 Jahre	5 (3,1 %)	0 (0,0 %)	0,388	5 (2,7 %)	59 (1,5 %)	0,108
40–59 Jahre	36 (22,6 %)	4 (17,4 %)	0,570	40 (22,0 %)	374 (9,7 %)	< 0,001^*^
60–79 Jahre	53 (33,3 %)	7 (30,4 %)	0,728	60 (33,0 %)	1311 (34,0 %)	0,874
≥ 80 Jahre	65 (40,9 %)	11 (47,4 %)	0,723	76 (41,8 %)	2050 (53,1 %)	0,003^*^
*Schwangerschaft*						
Ja	2 (1,3 %)	0 (0,0 %)	–	2 (1,1 %)	0 (0,00 %)	–
*Komorbidität*						
Bösartiger Tumor	15 (9,4 %)	5 (21,7 %)	0,143	20 (11,0 %)	1268 (32,8 %)	0,001^*^
Demenz	16 (10,1 %)	7 (30,4 %)	0,013^*^	23 (12,6 %)	569 (14,7 %)	0,434
*ECMO-Therapie*						
Ja	13 (8,2 %)	0 (0,0 %)	–	13 (7,1 %)	k. A.	–

*Min.* Minimum, *Max.* Maximum, *ECMO* extrakorporale Membranoxygenierung, *k.* *A.* keine Angabe

*p < 0,05

COVID-19-Verstorbene wiesen mit 59,1 % einen etwas höheren Männeranteil als Verstorbene ohne COVID-19-Erkrankung auf (50,8 %). Auch war das mittlere Sterbealter der COVID-19-Patienten im Vergleich zu Personen ohne SARS-CoV-2-Infektion deutlich jünger (71,9 vs. 76,6 Jahre, nicht dargestellt). 2 COVID-19-erkrankte Frauen verstarben während ihrer Schwangerschaft an der Viruskrankheit. In 13 Fällen (8,2 %) war eine ECMO-Behandlung bei COVID-19-Patienten eingeleitet worden.

### Sterbeorte von COVID-19-Patienten

**Figure 3:**
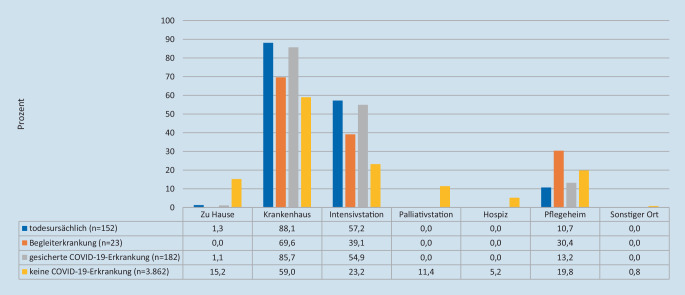

COVID-19-Erkrankte verstarben mit Abstand am häufigsten im Krankenhaus (Abb. 3). Patienten, bei denen die Krankheit todesursächlich war, verstarben in 88,1 % der Fälle im Krankenhaus, zu 10,7 % im Pflegeheim und zu 1,3 % zu Hause. Über die Hälfte aller im Krankenhaus verstorbenen Patienten (57,2 %) war auf einer Intensivstation verstorben. Palliativstationen oder Hospize spielten bei COVID-19-Patienten als Sterbeort keine Rolle. Personen ohne SARS-CoV-2-Infektion verstarben im Vergleich zu COVID-19-Patienten seltener im Krankenhaus auf einer Allgemein- oder Intensivstation und häufiger im Pflegeheim, zu Hause, auf einer Palliativstation, im Hospiz oder an einem sonstigen öffentlichen Ort.

**Figure 4:**
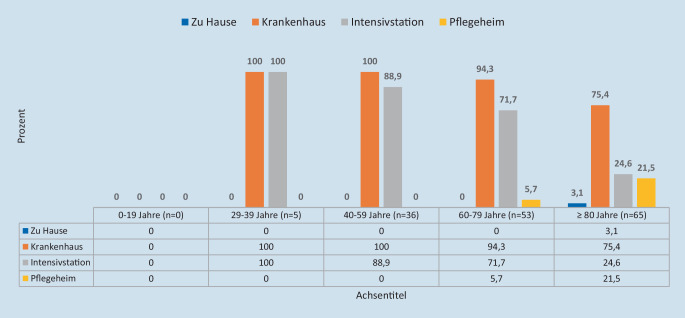

Stratifiziert nach Altersgruppen zeigte sich, dass sämtliche COVID-19-Patienten < 60 Jahre im Krankenhaus verstorben waren (Abb. 4). Alle Patienten < 40 Jahre waren auf einer Intensivstation verstorben. Mit zunehmendem Alter sank der Anteil dort Verstorbener moderat, bei über 79-Jährigen deutlich. Andererseits verstarben ältere COVID-19-Patienten häufig auch im Pflegeheim. Hier lag der Anteil in der Alterskategorie 80 Jahre und älter bei 21,5 %. Zu Hause verstarben lediglich betagte Menschen ≥ 80 Jahre (zu 3,1 %).

Tab. 2 gibt einen Überblick stratifiziert nach Sterbeort zu verstorbenen COVID-19 Patienten und deren Charakteristika (Tab. 2). Demnach verstarben jüngere Patienten primär im Krankenhaus und 2 ältere Personen zu Hause. Im Pflegeheim verstorbene COVID-19-Patienten wurden in 58,8 % der Fälle am Lebensende durch ein SAPV-Team betreut.

**Table 2:** 

	Zu Hause, *n* = 2		Krankenhaus, *n* = 156		Intensivstation, *n* = 100		Pflegeheim, *n* = 24	
Parameter	Todesursächlich	Begleiterkrankung	Todesursächlich	Begleiterkrankung	Todesursächlich	Begleiterkrankung	Todesursächlich	Begleiterkrankung
	*n* = 2 (100 %)	*n* = 0 (100 %)	*n* = 140 (100 %)	*n* = 16 (100 %)	*n* = 91 (100 %)	*n* = 9 (100 %)	*n* = 17 (100 %)	*n* = 7 (100 %)
*Geschlecht*								
Männlich	1 (50,0 %)	0 (0,0 %)	88 (62,9 %)	10 (62,5 %)	61 (67,0 %)	7 (77,8 %)	5 (29,4 %)	2 (28,6 %)
Weiblich	1 (50,0 %)	0 (0,0 %)	52 (37,1 %)	6 (37,5 %)	30 (33,0 %)	2 (22,2 %)	12 (70,6 %)	5 (71,4 %)
*Sterbealter (Jahre)*								
Mittelwert	91,7	–	69,9	66,2	63,9	61,4	85,2	90,2
Median	91,7	–	72,3	71,2	63,4	66,4	85,7	87,1
Min./Max.	83,7/99,6	–	28,6/101,3	4,6/89,6	28,6/91,3	4,6/89,6	72,2/97,0	84,6/99,8
*Altersgruppen*								
0–9 Jahre	0 (0,0 %)	0 (0,0 %)	0 (0,0 %)	1 (6,3 %)	0 (0,0 %)	1 (11,1 %)	0 (0,0 %)	0 (0,0 %)
10–19 Jahre	0 (0,0 %)	0 (0,0 %)	0 (0,0 %)	0 (0,0 %)	0 (0,0 %)	0 (0,0 %)	0 (0,0 %)	0 (0,0 %)
20–29 Jahre	0 (0,0 %)	0 (0,0 %)	2 (1,4 %)	0 (0,0 %)	2 (2,2 %)	0 (0,0 %)	0 (0,0 %)	0 (0,0 %)
30–39 Jahre	0 (0,0 %)	0 (0,0 %)	3 (2,1 %)	0 (0,0 %)	3 (3,3 %)	0 (0,0 %)	0 (0,0 %)	0 (0,0 %)
40–49 Jahre	0 (0,0 %)	0 (0,0 %)	14 (10,0 %)	1 (6,3 %)	12 (13,2 %)	0 (0,0 %)	0 (0,0 %)	0 (0,0 %)
50–59 Jahre	0 (0,0 %)	0 (0,0 %)	22 (15,7 %)	3 (18,8 %)	20 (22,0 %)	2 (22,2 %)	0 (0,0 %)	0 (0,0 %)
60–69 Jahre	0 (0,0 %)	0 (0,0 %)	24 (17,1 %)	3 (18,8 %)	20 (22,0 %)	3 (33,3 %)	0 (0,0 %)	0 (0,0 %)
70–79 Jahre	0 (0,0 %)	0 (0,0 %)	26 (18,6 %)	4 (25,0 %)	18 (19,8 %)	2 (22,2 %)	3 (17,6 %)	0 (0,0 %)
80–89 Jahre	1 (50,0 %)	0 (0,0 %)	36 (25,7 %)	4 (25,0 %)	15 (16,5 %)	1 (11,1 %)	11 (64,7 %)	4 (57,1 %)
≥ 90 Jahre	1 (50,0 %)	0 (0,0 %)	13 (9,3 %)	0 (0,0 %)	1 (1,1 %)	0 (0,0 %)	3 (17,6 %)	3 (42,9 %)
*Begleiterkrankungen*								
Bösartiger Tumor	0 (0,0 %)	0 (0,0 %)	14 (10,0 %)	5 (31,3 %)	8 (8,8 %)	3 (33,3 %)	1 (5,9 %)	0 (0,0 %)
Demenz	0 (0,0 %)	0 (0,0 %)	4 (2,9 %)	2 (12,9 %)	1 (1,1 %)	1 (11,1 %)	12 (70,6 %)	5 (71,4 %)
*ECMO-Therapie*								
Ja	–	–	13 (9,3 %)	0 (0,0 %)	13 (14,3 %)	0 (0,0 %)	–	–
*SAPV-Betreuung*								
Ja	0 (0,0 %)	0 (0,0 %)	0 (0,0 %)	0 (0,0 %)	0 (0,0 %)	0 (0,0 %)	10 (58,8 %)	6 (85,7 %)

*Min.* Minimum, *Max.* Maximum, *ECMO* extrakorporale Membranoxygenierung, *SAPV* spezialisierte ambulante Palliativversorgung

Im Kontext der zeitlichen Entwicklung der Sterbeorte zeigte sich bei Tumor- und Demenzerkrankungen im Vergleich zum letzten Untersuchungsjahr 2017 keine relevante prozentuale Veränderung des Sterbeortes zu Hause (siehe Tabellen Z1, Z2 im Onlinematerial).

## Diskussion

In der vorliegenden Studie ließ sich nachweisen, dass Patienten mit einer tödlich verlaufenden COVID-19-Erkrankung in der großen Mehrzahl (88,1 %) im Krankenhaus verstarben. Dabei ereigneten sich über die Hälfte aller Sterbefälle auf einer Intensivstation. Todesfälle im Pflegeheim traten mit einer Häufigkeit von 10,7 % auf, nur 1,3 % der Erkrankten verstarben zu Hause. Palliativstationen und Hospize spielten als Sterbeort keine Rolle.

Im Rahmen der Studie wurden 4044 Todesbescheinigungen des Jahres 2021 in der Stadt Münster ausgewertet. Insgesamt verstarben 182 Personen an oder mit einer COVID-19-Erkrankung, entsprechend einem Anteil von 4,5 % gemessen an der Zahl sämtlicher Sterbefälle. Im selben Jahr wurden deutschlandweit insgesamt 73.306 COVID-19-Todesfälle dem Robert Koch-Institut gemeldet [30]. Wenn diese Fälle ins Verhältnis zur Anzahl der bundesweiten Sterbefälle von 1,02 Mio. gesetzt werden, lässt sich ein Anteil von 7,2 % ableiten [31]. Im Vergleich traten 2021 in der Stadt Münster weniger COVID-19-Sterbefälle auf als im gesamten Bundesgebiet.

Bei 159 in der Stadt Münster verstorbenen COVID-19-Patienten war der Tod allein auf die Viruserkrankung zurückzuführen (3,9 %), was einem Anteil von 87,4 % bezogen auf alle in den Todesbescheinigungen dokumentierten COVID-19-Erkrankungsfälle entspricht. Diese Quote deckt sich mit Daten des COVID-19-Autopsieregisters (DeRegCOVID). Demzufolge verstarben von den im Zusammenhang mit COVID-19 obduzierten Personen 86 % an der Erkrankung, während bei 14 % COVID-19 als Begleiterkrankung gewertet wurde [32].

Im Beobachtungsjahr 2021 wurden im westfälischen Studiengebiet mittels PCR-Testung 10.858 Neuinfektionen nachgewiesen [29]. Ende Dezember 2020 hatte sich in einem Seniorenheim ein schwerer COVID-19-Ausbruch ereignet, in dessen Verlauf insgesamt 47 infizierte Personen und 8 Todesfälle zu beklagen waren [33]. Zudem wurden aufgrund des Vorhaltens von ECMO-Zentren an 3 Krankenhäusern der Maximal- und Schwerpunktversorgung im Jahr 2021 mehrere Sekundärverlegungen schwerstkranker COVID-19-Patienten nach Münster durchgeführt [18]. Dabei wurden die Patienten sowohl aus stark betroffenen deutschen Pandemiegebieten als auch aus den benachbarten Niederlanden und Belgien eingeflogen. Über die Letalität bei diesen Patienten liegt den Autoren keine genaue Information vor, die Letalität dürfte jedoch als sehr hoch einzuschätzen sein.

Die Ergebnisse unserer Studie zu den Sterbeorten ähneln einer Untersuchung von Gleich et al., die eine Auswertung von Todesbescheinigungen mit Fokus auf die Todesursachen Influenza und COVID-19 durchführten [22]. Analysiert wurden die Todesbescheinigungen aller 12.441 Sterbefälle der Stadt München im Zeitraum vom 01.03. bis 31.12.2020. Von den 1029 gesicherten COVID-19-Todesfällen ereigneten sich 71,8 % im Krankenhaus, 24,4 % in einer stationären Pflegeeinrichtung und 3,8 % zu Hause. Der Anteil der auf Intensivstationen Verstorbenen unterschied sich jedoch deutlich: Mit 28,7 % lag er in München nur halb so hoch wie in unserer Studie (54,9 %). Ein zentraler Erklärungsansatz wäre, dass strukturelle gesundheitsmedizinische Angebote hier den Sterbeort mitbestimmten („induzierte Nachfrage“; [20]). So weisen laut DIVI-Intensivregister beide Städte jeweils 3 Kliniken mit ECMO-Behandlungsplätzen auf, bei einer 5‑mal höheren Einwohnerzahl von München gegenüber Münster [16]. Zudem lag das Sterbealter mit 81,7 Jahren gut 10 Jahre höher als in der vorliegenden Untersuchung. Ob dies ein weiterer Grund für die selteneren intensivstationären Behandlungen war, lässt sich aus den Daten der Münchener Studie nicht ablesen. Es kann jedoch spekuliert werden, dass ältere COVID-19-Patienten im Vergleich zu jüngeren aufgrund eines schlechteren individuellen Gesundheitsprofils (höhere Wahrscheinlichkeit für Multimorbidität, eingeschränkter Karnofsky-Index) seltener intensivmedizinisch behandelt wurden, da ihre Überlebenswahrscheinlichkeit von ärztlicher Seite als zu schlecht eingestuft wurde. Auch könnte die Existenz einer Patientenverfügung – welche eher bei älteren als bei jüngeren Patienten vorzufinden ist – mit festgelegtem DNR/DNI-Status (Do Not Resuscitate/Do Not Intubate – Verzicht auf Wiederbelebung) der Verlegung auf eine Intensivstation entgegengestanden haben. Diese Aspekte könnten auch für unsere Studienbeobachtung als mögliche Erklärungsansätze herangezogen werden. So stellten wir fest, dass junge verstorbene COVID-19-Patienten < 60 Jahre zu 100 % am Lebensende auf einer Intensivstation behandelt worden waren, hingegen betagte Patienten über 80 Jahre nur in 24,6 % der Fälle.

Eine weitere Sterbeortuntersuchung führten Behnke et al. im Landkreis Gießen anhand 471 gesicherter COVID-19-Todesfälle durch [23]. Der Beobachtungszeitraum erstreckte sich von November 2020 bis April 2021 und erfasste damit Sterbefälle der zweiten und dritten Pandemiewelle. Todesfälle im Krankenhaus machten mit 63,5 % einen etwas niedrigeren und Sterbefälle im Pflegeheim mit 34,0 % einen etwas höheren Anteil aus als in unseren Studienergebnissen. Der Sterbeort Krankenhaus wurde noch weiter differenziert: Bezogen auf alle untersuchten 471 Sterbefälle verstarben 32,2 % der Patienten auf einer Intensivstation, 21,1 % auf einer Allgemeinstation und 1,3 % an einem sonstigen Ort im Krankenhaus. Mit 2,1 % waren Sterbefälle zu Hause ebenso selten wie in unserer Untersuchung mit 1,1 %.

Internationale Studien zum Sterbeort verstorbener COVID-19-Patienten beschreiben ähnliche Prozentzahlen. So wurde im Vereinigten Königreich im Zeitraum von 03/2020 bis 03/2021 der Sterbeort von insgesamt 147.282 verstorbenen COVID-19-Patienten analysiert. Hierbei verstarben 68 % im Krankenhaus, 24 % im Pflegeheim, 6 % zu Hause, 1 % in Hospizen und 1 % an sonstigen Orten [34]. Daten aus den USA belegen, dass von den bis Ende Oktober 2022 ca. 1,06 Mio. Verstorbenen 66,7 % im Krankenhaus verstarben, 14,6 % im Pflegeheim, 9,6 % zu Hause, 3,3 % in Hospizen und 5,8 % an einem sonstigen Ort [35]. Zu dem höheren Anteil zu Hause Verstorbener gehörten in einer Erhebung zum Jahr 2020 viele jüngere Menschen < 50 Jahren. Die Autoren ziehen als mögliche Begründung in Betracht, dass viele junge Erkrankte keinen ausreichenden Krankenversicherungsschutz hatten und Angst vor den Krankenhauskosten bestand [36].

Die hohe Zahl der im Krankenhaus und insbesondere auf Intensivstationen verstorbenen Patienten legt nahe, dass die Behandlung initial auf Heilung ausgerichtet war. Atemnot, das vorrangigste Symptom einer schweren COVID-19-Erkrankung, kann in Extremsituationen bei betroffenen Patienten und Angehörigen existentielle Ängste vor einem qualvollen Tod auslösen [37]. In diesem Kontext dürfte auch der sich schnell in eine kritische Richtung entwickelnde Krankheitsverlauf eine wichtige Rolle gespielt haben. So weisen epidemiologische Daten auf eine kurze Zeitspanne zwischen Symptombeginn und Versterben bei fulminanten Verläufen hin. Diese lag im Mittel bei gerade einmal 15,9 Tagen (95 %-KI: 13,1–18,8 Tage; [38]). Daher könnte auch für Menschen, die aufgrund einer anderen fortgeschrittenen Erkrankung oder hohen Lebensalters unter anderen Umständen keinen Krankenhausaufenthalt mehr gewünscht hätten, die Hoffnung auf Überleben und Heilung sowie der Wunsch auf bestmögliche Behandlung, Überwachung und Symptomlinderung im Krankenhaus im Vergleich zu ambulanten Therapiemöglichkeiten ausschlaggebend für eine stationäre Aufnahme gewesen sein. Die Tatsache, dass in Münster lediglich 10,7 % der COVID-19-Patienten in einer stationären Pflegeeinrichtung und gerade einmal 1,3 % zu Hause verstarben, untermauert diese Hypothese.

Einen Einfluss auf den Sterbeort dürften auch gesetzliche Vorgaben zum Infektionsschutz ausgeübt haben. Wenngleich in den analysierten Todesbescheinigungen auch mehrfach ein palliatives Therapiekonzept benannt wurde, verstarb niemand auf einer Palliativstation. Dies lag vermutlich daran, dass hospitalisierte COVID-19-Patienten zur Vermeidung nosokomialer Infektionsketten auf separaten Isolierstationen behandelt wurden [39]. Zudem wurden in stationären Pflegeeinrichtungen im Bedarfsfall Isolierungsmaßnahmen angeordnet. Trotz dessen waren nicht selten lokale COVID-19-Ausbrüche in Heimen zu verzeichnen. Dies wurde auch in der Stadt Münster Ende Dezember/Anfang Januar 2021 beobachtet [33]. Dass COVID-19-Patienten nicht in einem Hospiz verstarben, mag auch mit der Tatsache zusammenhängen, dass in dieser Institution vorrangig Tumorpatienten am Ende ihres Lebens betreut werden und die Wartezeit auf einen Hospizplatz in der Regel mehrere Wochen beträgt.

Bei den im Pflegeheim Verstorbenen zeigte sich in der vorliegenden Arbeit, dass 16 von 24 COVID-19-Patienten (66,7 %) am Lebensende eine spezialisierte ambulante Palliativbetreuung (SAPV) erhalten hatten. Dies deutet auf eine gute Vernetzung zwischen SAPV und Pflegeheimen in der Stadt Münster hin. Andererseits war bei den 2 häuslichen COVID-19-Sterbefällen kein SAPV-Team in die Patientenmitbetreuung eingebunden worden.

Im Gegensatz zu Daten aus dem Vereinigten Königreich, die in der Pandemie einen Anstieg der ambulanten Sterbefälle zeigten [24, 34], sah man in unserer Untersuchung keine relevante Veränderung bei den Sterbeorten außerhalb des Krankenhauses. Hier fokussierten wir uns speziell auf die Gruppe von Tumor- und Demenzerkrankten. Möglicherweise erklären Unterschiede im Leistungsangebot des britischen und deutschen Gesundheitssystems die beobachtete Differenz. So hatte Deutschland in den vergangenen Jahren mit einer Kapazität von 33,9 Betten/100.000 Einwohnern etwa 3‑mal so viele Intensivbetten vorzuweisen wie England mit 10,5/100.000 [40].

### Limitationen

Die Studie basierte auf ausgewerteten Todesbescheinigungen der Stadt Münster. Daher können die Studienergebnisse für Deutschland nicht als repräsentativ gelten. Wichtige Determinanten, welche neben dem Sterbeort auch das Infektions- und Krankheitsrisiko mitbestimmen, wurden im Totenschein nicht immer dokumentiert. Hierzu zählen u. a. die Schwere der Erkrankung, der Impfstatus, spezifische Risikofaktoren (u. a. Adipositas) sowie der Sozialstatus (Wohnsituation, familiäres Umfeld etc.).

## Fazit

Laut der Weltgesundheitsorganisation (WHO) ist nach knapp 3 Jahren das Ende der Pandemie in Sicht. Dennoch bleibt vor allem für ältere Menschen und sonstige vulnerable Personen ein erhöhtes Risiko, infolge einer SARS-CoV-2-Infektion zu versterben [41].

In dieser Sterbeortstudie der Stadt Münster konnte gezeigt werden, dass COVID-19-Patienten mit tödlichem Krankheitsverlauf im Jahr 2021 hauptsächlich im Krankenhaus verstarben. Hierbei verstarben Patienten < 60 Jahre zu 100 % auf einer Intensivstation, betagte Patienten ≥ 80 Jahre hingegen nur in 24,6 % der Fälle. Dies lässt darauf schließen, dass das individuelle Gesundheitsprofil eines Patienten und die sich hieraus ableitende Überlebensprognose den Sterbeort mitbestimmten. Stationäre Pflegeeinrichtungen spielten als Sterbeort bei lokalen Infektionsausbrüchen eine gewisse Rolle, der Sterbeort zu Hause nur sehr sporadisch. Sterbefälle von COVID-19-infizierten Personen auf einer Palliativstation oder im Hospiz wurden nicht beobachtet.

Weitere Studien sollten angestrebt werden, um insbesondere die Wirkung des Sterbeortes auf hinterbliebene Angehörige, Freunde und Bekannte verstorbener COVID-19-Patienten bezüglich des Abschiednehmens und der Trauer vertiefender zu untersuchen. Hier sollten Versorgungsdefizite erkannt und Handlungsempfehlungen abgeleitet werden, um in Hinblick auf mögliche zukünftige Pandemien hochinfektiöser Erreger eine adäquate Sterbebegleitung gewährleisten zu können.

## Supplementary Information




