# Supplementary material for: Places of death of COVID-19 patients: an observational study based on evaluated death certificates from the city of Muenster, Germany (2021)
Source: Bundesgesundheitsblatt Gesundheitsforschung Gesundheitsschutz. 2023 May 26;66(9):962–71. [Article in German] doi: 10.1007/s00103-023-03702-7 (PMC10214335; doi:10.1007/s00103-023-03702-7)
Supplement: Supplementary file 1 [file 103_2023_3702_MOESM1_ESM.pdf]

Onlinematerial, Tab. Z1. Zeitlicher Trend bei den Sterbeorten von Tumorpatienten (2001 – 2021)

	**2001**	**2011**	**2017**	**2021**	***p***
	**N = 1.006**	**N = 1.252**	**N = 1.295**	**N = 1.288**	*2017 vs. 2021*
Zu Hause	172 (17,1 %)	282 (22,5 %)	189 (14,6 %)	198 (15,4 %)	0,582
Krankenhaus	685 (68,1 %)	677 (54,1 %)	746 (57,6 %)	780 (60,6 %)	0,129
Palliativstation	0 (0,0 %)	68 (5,4 %)	156 (12,0 %)	314 (24,4 %)	0,001*
Hospiz	104 (10,3 %)	192 (15,3 %)	234 (18,1 %)	186 (14,4 %)	0,014*
Pflegeheim	43 (4,3 %)	100 (8,0 %)	125 (9,7 %)	124 (9,6 %)	0,999
Sonstiger Ort	2 (0,2 %)	1 (0,1 %)	1 (0,1 %)	0 (0,0 %)	-

* $p < 0{,}05$

Onlinematerial, Tab. Z2. Zeitlicher Trend bei den Sterbeorten von Demenzerkrankten (2001 – 2021)

	**2001**	**2011**	**2017**	**2021**	***p***
	**N = 152**	**N = 341**	**N = 493**	**N = 592**	*2017 vs. 2021*
Zu Hause	41 (27,0 %)	55 (16,1 %)	60 (12,2 %)	72 (12,2 %)	0,999
Krankenhaus	69 (45,4 %)	103 (30,2 %)	113 (22,9 %)	116 (19,6 %)	0,204
Palliativstation	0 (0,0 %)	3 (0,9 %)	7 (1,4 %)	11 (1,9 %)	0,639
Hospiz	1 (0,7 %)	7 (2,1 %)	6 (1,2 %)	10 (1,7 %)	0,618
Pflegeheim	41 (27,0 %)	176 (51,6 %)	314 (63,7 %)	394 (66,6 %)	0,337
Sonstiger Ort	0 (0,0 %)	0 (0,0 %)	0 (0,0 %)	0 (0,0 %)	-

* $p < 0{,}05$
